# Lesion of the hip abductor mechanism

**DOI:** 10.1051/sicotj/2016020

**Published:** 2016-07-06

**Authors:** Horacio Caviglia, Guillermo Cambiaggi, Nosrat Vattani, María Eulalia Landro, Gustavo Galatro

**Affiliations:** 1 Orthopaedic and Traumatology Department, General Hospital Dr. Juan A. Fernández Buenos Aires Argentina

**Keywords:** Hip, Revision surgery, Abductor muscles, Injury, Biomechanics

## Abstract

*Introduction*: The disruption of the abductor muscles of the hip after hip revision surgery often causes limping, pain, and instability of the implant. The purpose of our paper is to describe a mesh technique to repair hip abductor mechanism injuries after hip revision.

*Patients and methods*: Forty-six patients with hip abductor damage after prosthetic revision were treated. Inclusion criteria were: patients presenting with prosthetic loosening, complaint of pain, and with a positive Trendelenburg sign due to deficient abductor muscle mechanisms. Thirty-one were women (67.39%) with an average age of 64 years (34–82 years). The number of previous revision surgeries was three (two to seven). The Merle d’Aubigné score and variants before and after treatment were also reported.

*Results*: In the postoperative follow-up after hip revision with the mesh technique, the Merle d’Aubigné score improved and the Trendelenburg sign was negative in 78.3% of the patients (*p* < 0.001). Also, the Trendelenburg test with the knee flexed was negative in 60.9% (*p* < 0.001) and the stair-climbing test was negative in 60.9% of cases (*p* < 0.001). The gluteus medius test in the lateral position was negative in 52.2% of patients, and in the lateral position with the knee flexed it was negative in 47.8% of patients (*p* < 0.001).

*Discussion*: Repair of the abductor mechanism with the mesh technique has proven effective for both partial and total lesions.

## Introduction

Hip abductor muscles are often damaged during both Charnley’s approach with trochanteric osteotomy and during Hardinge's approach [1–5]. The abductor muscles may be severely torn due to multiple surgeries, infection, and chronic inflammation [6]. The partial or total disruption of the abductor muscles of the hip after hip revision surgery often causes limping and pain and contributes to instability of the implant [7]. The incidence has been reported between 0.08 and 22% [8], higher in women [9, 10] and is especially high in the elderly [11].

Other common causes of abductor muscle injury are bone stock defects due to multiple hip revision surgery or osteolysis caused by polyethylene or metal debris [12, 13]. Lachiewicz [14] describes three causes of abductor muscle ruptures: degenerative or traumatic tears associated with the surgical approach for arthroplasty in femoral fractures or osteoarthritis and avulsion or repair failure as a result of anterolateral or transgluteal surgical approach. Abductor muscles may also be damaged during the anterolateral surgical approach producing a neurapraxia or severing of the superior gluteal nerve [15]. Gabrion [16] described three cases of rupture of the gluteus medius associated with hip osteoarthritis.

The purpose of this paper is to describe and evaluate the technique we use to repair hip abductor mechanism injuries and rupture with a mesh technique using Prolene^®^ (©Ethicom US, Johnson & Johnson, NJ, USA) regardless of the reconstruction prosthetic acetabular and femoral components. The uniqueness of this treatment is to maintain the continuity between the *gluteus medius* and the *vastus lateralis* tightening the *fascia lata* by the mesh without fixing it to the femur.

We also propose to further classify the type of injury to the abductor apparatus according to the size and location of the lesion and to correlate it with the degree of success of the repair with our technique.

## Patients and methods

We treated 46 patients with a loose hip prosthesis, associated with injury of hip abductor mechanism, between 2002 and 2012 in our Institution. The inclusion criteria were: patients complained of pain, associated prosthetic loosening for both acetabular and femoral components, and abductor mechanism damage. Patients with neurologic damage of the abductor mechanism were not included.

Thirty-one were women (67.39%), the average age was 64 years (34–82). The number of previous revisions was three (two to seven). Eleven patients had had two previous revisions (23.9%), fourteen patients had had three previous procedures (30.4%), nine patients had had four revisions (19.6%), eight patients had had five revisions (17.4%), and four patients had had six revisions (8.7%) (Table 1). The average follow-up was six years (two to twelve).

**Table 1.** Revisions prior to this treatment.

Revisions	Frequency (%)
2	11 (23.9)
3	14 (30.4)
4	9 (19.6)
5	8 (17.4)
6	4 (8.7)
Total	46

In the preoperative evaluation, the Trendelenburg sign was positive in 100% of patients and the Trendelenburg test with the knee flexed was positive in 100%, stair-climbing test was positive in 100% of cases. The *gluteus medius muscle* test in the lateral position was positive in 100% of patients, and in the lateral position with the knee flexed it was positive in 100% of patients.

Both components, acetabular and femoral, were revised. Patients were classified at the time of hip revision.

### Classification

**Figure 1.**
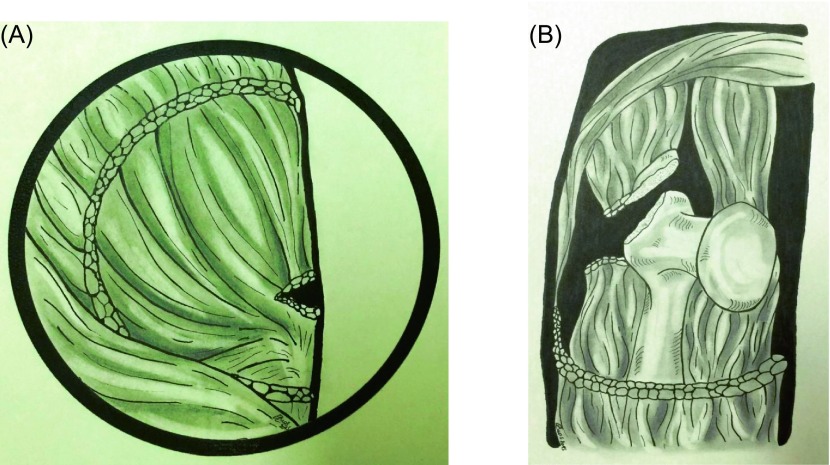
Lesions were classified intra-operatively. (A) Types I and III: the abductor mechanism has partial rupture with trochanteric displacement (<2.5~cm). (B) Types II and IV: the abductor mechanism is discontinuous (total rupture) with a trochanteric displacement (>2.5~cm).

Patients were classified intra-operatively. First we determined whether the abductor apparatus had partial or total rupture (the *gluteus medius muscle* and *vastus lateralis muscle* were attached to each other). With a digital caliper (Mitutoyo^®^) the distance between the broken piece disruptured ends of the greater trochanter and its bed with the hip extended and from the vertical center of the femur was measured. The lesions were classified as follows: type I lesions, the abductor mechanism has partial rupture with trochanteric displacement <2.5 cm (seven patients, 15.2%); type II lesions, the abductor mechanism has partial rupture with a trochanteric displacement >2.5 cm (six patients, 13%); type III lesions, the abductor mechanism is discontinuous (total rupture) with a trochanteric displacement <2.5 cm (17 patients, 37%); type IV lesions, the abductor mechanism is discontinuous with a trochanteric displacement >2.5 cm (13 patients, 28.3%) (Figure 1). Type V lesions, the abductor mechanism is continuous but displaced onto the femur (three patients, 6.5%) (Table 2).

**Table 2.** Classification of the abductor muscles lesion.

Classification	Frequency
I	7 (15.2)
II	6 (13.0)
III	17 (37.0)
IV	13 (28.3)
V	3 (6.5)
Total	46

We had considered a gap limit of 2.5 cm, that is the maximum distance that allows use of the mesh with a direct suture with additional release to bring the terminal edges of the *gluteus medius*.

This technique did not fix the *gluteus medius* to the greater trochanter, we divided the patients into two groups, based on the separation of the edges of the abductor mechanism. We divide the study population into: group A, included 24 patients with displacement smaller than 2.5 cm (types I and III), and group B, included 19 patients with displacements larger than 2.5 cm (types II and IV).

**Figure 2.**
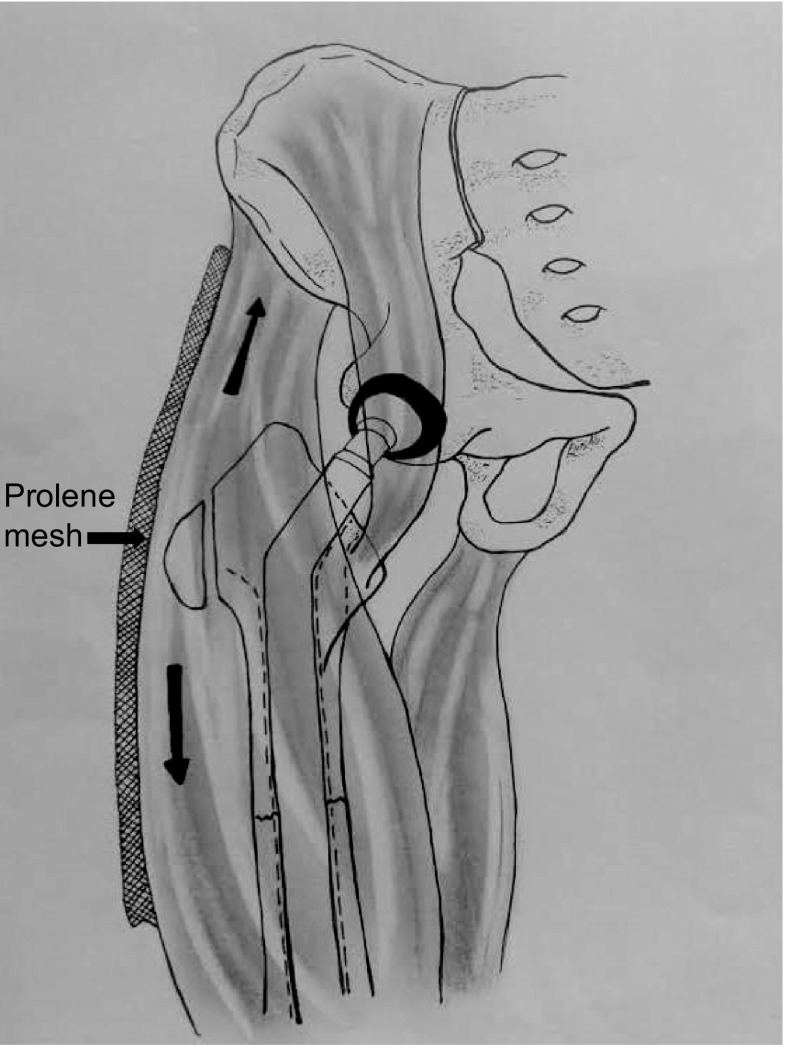
Repair of the abductor mechanism was performed with mesh technique (Prolene^®^ ©Ethicom, Johnson & Johnson, NJ, USA).

Repair of the abductor mechanism was performed with the mesh technique (Prolene^®^ ©Ethicom, Johnson& Johnson, NJ, USA) (Figure 2).

When the *gluteus medius muscle* was attached, in types I and II (partial rupture), repairs were easier, because the *gluteus medius* insertion is still attached by an area of fibrosis. The mesh was placed in a fan shape on the proximal *gluteus medius muscle* and fixed by continuous points on the edge (performing it as surget), front and back, and through separate point on the top edge. Subsequently the area of fibrosis was resected, to meet the edges and the limb was raised, to reduce the tension of the muscles that will be approximated. The mesh was folded and placed on the *vastus lateralis muscle* and the tensor fascia lata was sutured with a continuous suture, performing it as surget. The distal length of the mesh should be between 15 and 20 cm.

When the abductor muscle system was detached in types III and IV (total rupture), the first step was to locate the *gluteus medius muscle*, which was proximally retracted. The *gluteus medius muscle* must be released and put back in the lateral position protecting the blood vessels. A fan-shaped piece of Prolene^®^ mesh (©Ethicom, Johnson & Johnson, NJ, USA) was inserted into place and sutured along three of its four edges with a continuous suture. The distal edge was then folded two or four times to settle it on the *vastus lateralis muscle*. Subsequently the limb was raised and the mesh tensioned to reposition the *gluteus medius muscle* distally. Finally, each edge of the mesh was sutured to the *vastus lateralis muscle* and the tensor fascia lata, with a continuous suture, performing it as surget. The distal length of the mesh should be between 15 and 20 cm.

**Figure 3.**
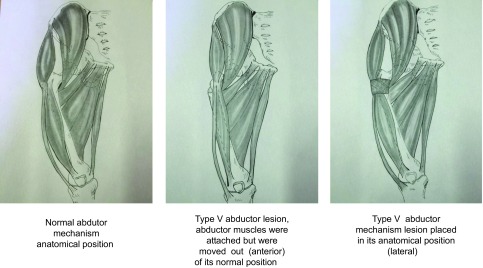
Type V abductor injury occurred when the abductor muscles were attached but were moved out of their normal position.

Type V abductor injury occurred when the abductor muscles were attached but were moved out of its normal position (Figure 3).

Avoid harm to blood vessels when resecting fibrosis. Once we have located the anterior edge of the *gluteus medius* and *vastus lateralis muscles*, a 30 × 30 cm piece of Prolene^®^ mesh (©Ethicom, Johnson & Johnson, NJ, USA) was sutured to it by a continuous suture of Prolene^®^ (Johnson & Johnson, NJ, USA), thread 5 cm from the free edge of the mesh.

It is not necessary to tense the abductor apparatus to return it to its original position as it was already shortened. The lower limb should be placed in abduction and the mesh is pulled laterally in order to put the abductor muscles back in place. The free edge of the mesh is sutured to the aponeurotic fascia and then reinforced with the same suture.

The excess material is trimmed with mesh scissors. Subsequently the free flap above the mesh is fixed to the anterior face of the deep fascia, thus anchoring the abductor muscles in their normal anatomic position.

Patients undergoing rehabilitation after repair were generally able to sit or stand, but body weight loading must not exceed 25% during the first two weeks and 50% during the next four weeks. Abduction exercises begin after four weeks. In those cases where revisions were complex, rehabilitation times vary.

### Statistical analysis

We carried out a descriptive statistical analysis of each parameter analyzed.

Homoscedasticity was evaluated with the Levene Test and normality with Kolmogorov-Smirnov and Shapiro Wilks tests.

To describe the quantitative variables average, standard deviation, median, minimum, and maximum were calculated. Kolmogorov-Smirnov and Student’s *t*-tests were used for the comparison of groups.

The McNemar test for paired samples was used to compare the percentages of negative Trendelenburg signs.

In all cases, statistical test for independent samples and significance levels of <5% were applied to reject the null hypothesis.

## Results

Before the surgery, the Merle d’Aubigné score was 6.89 (4–10) and all patients had positive Trendelenburg sign. The Merle d’Aubigné score improved an average of 14.7 (12–17) points postoperatively (*p* < 0.001).

**Figure 4.**
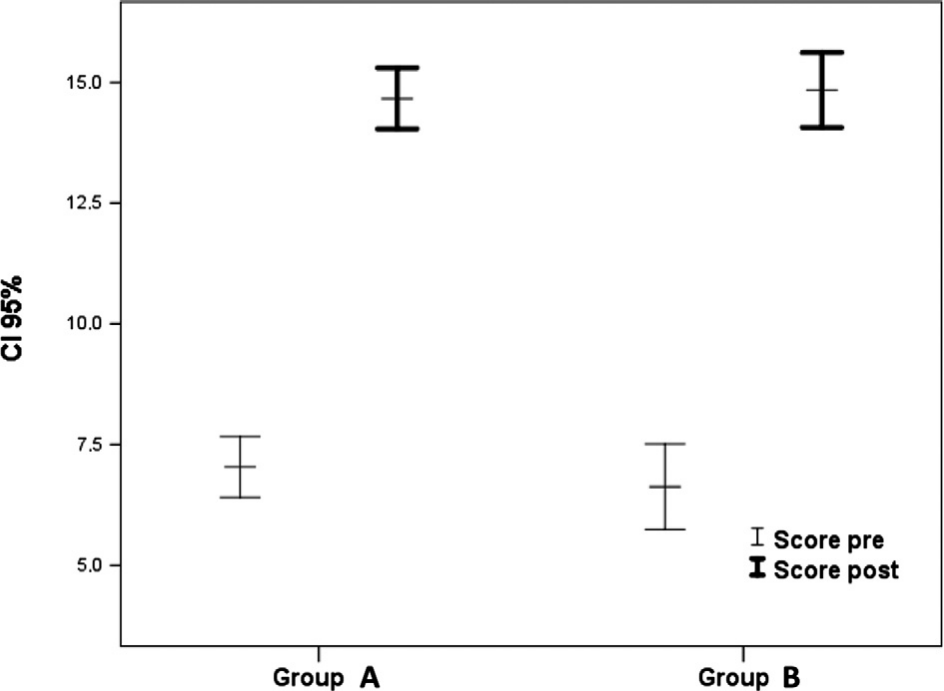
Merle d’Aubigné score pre- and post-surgery. Group A (<2.5 cm) vs. group B (>2.5 cm).

There were no statistical differences between the groups A and B (< or > than 2.5 cm of displacement) for the Merle d’Aubigné score (Figure 4).

**Figure 5.**
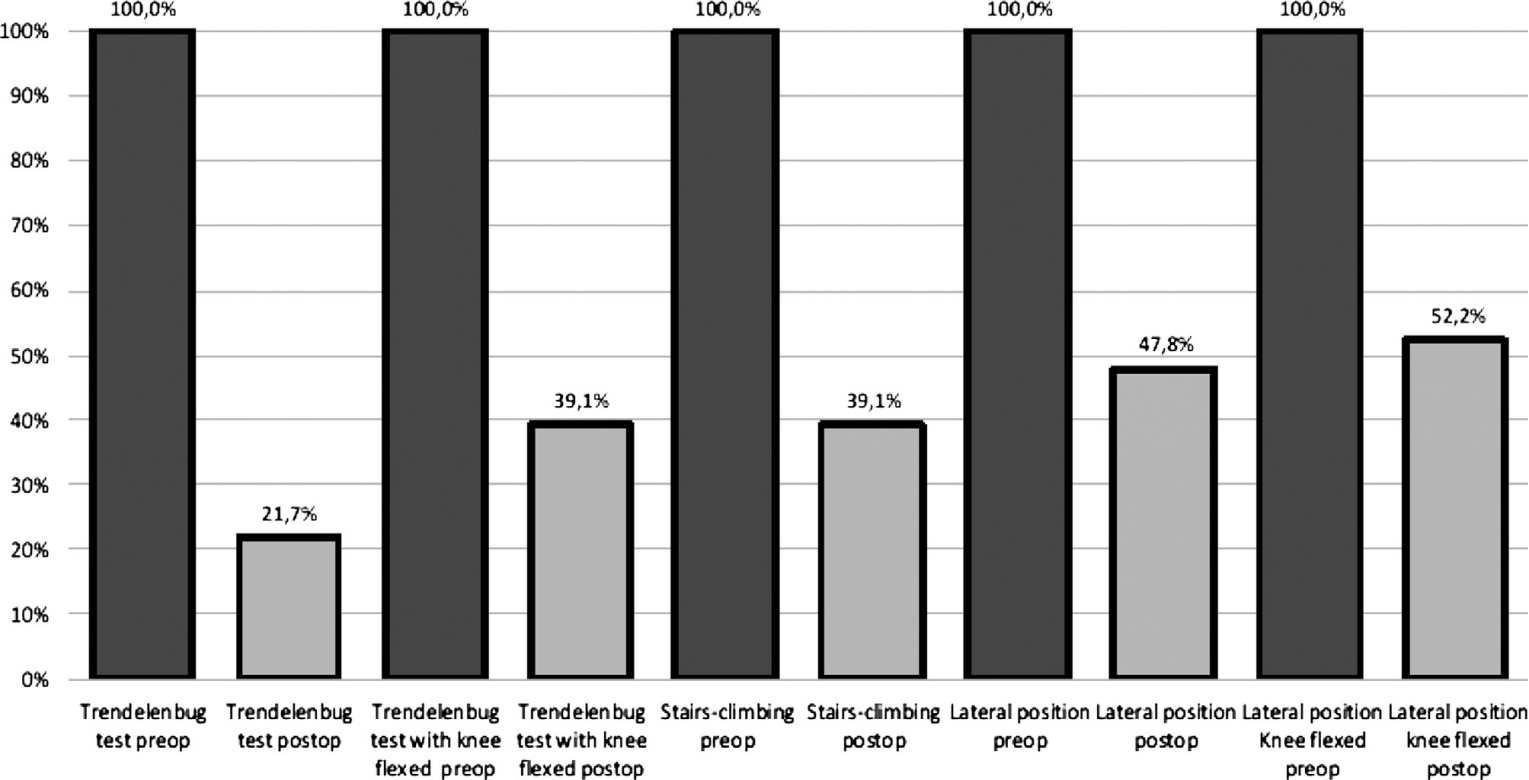
Trendelenburg test and variants scores before (preop) and after (postop) surgery.

In the postoperative evaluation, the Trendelenburg sign was negative in 78.3% of patients (*p* < 0.001) and the Trendelenburg test with the knee flexed was negative in 60.9% (*p* < 0.001), stair-climbing test was negative in 60.9% of cases (*p* < 0.001). The *gluteus medius muscle* test in the lateral position was negative in 52.2% of patients, and in the lateral position with the knee flexed it was negative in 47.8% of patients (*p* < 0.001) (Figure 5).

**Figure 6.**
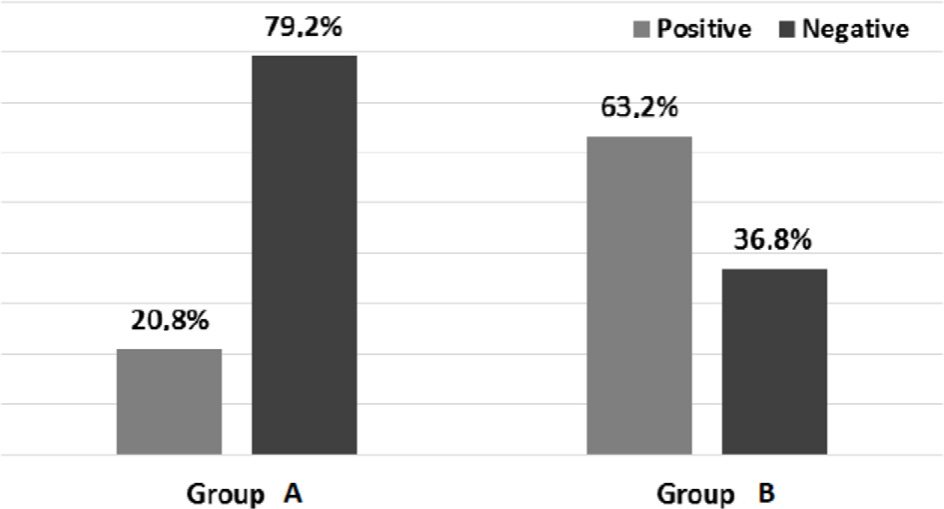
Stair-climbing test after surgery. Group A (<2.5 cm) vs. group B (>2.5 cm). (*p* < 0.0048*).

In group A, displacement less than 2.5 cm, 75% of the patients had no Trendelenburg sign and in group B, displacement larger than 2.5 cm, 42.1% of the cases had negative Trendelenburg sign (*p* < 0.028). There were also significant differences in the postoperative test ascending stairs: 79.2% of the patients were negative in group A and 36.8% of the group B (*p* < 0.0048) (Figure 6).

## Discussion

Hip abductor injury after hip replacement leads to limping, difficulty walking, and, in many cases, prosthetic instability. In this article, we propose a technique developed in our center to treat the abductor mechanism injury with an associated loose hip prostheses. The loose prostheses were revised and the abductor mechanism was repaired with a mesh technique in which there is plenty of experience in general surgery for soft tissue and hernia repair [17–19].

Our study basically assesses the functional recovery of abductor mechanism, regardless of reconstruction prosthetic acetabular and femoral components.

We have used the Prolene mesh for proximal femoral bone reconstruction instead of metal mesh for more than 20 years, and to repair the abductor mechanism for the last 13 years [20, 21].

The aim of this treatment is simply to maintain the continuity between the *gluteus medius* and the *vastus lateralis,* tightening the *fascia lata* by the mesh, minimizing the possible gap between them, and without setting this reconstruction to the greater trochanter.

The clinical outcome of this treatment has been successful, improving the Merle d’Aubigné score. We did not see a difference in the clinical outcome of Merle d’Aubigné score between groups A and B, with a gap distance more or less than 2.5 cm. This is probably due to the score of Merle d’Aubigné being used to evaluate gait, and here we will not find differences between both groups, as the lower limb is extended and flat on the floor.

We have elected to use the 2.5 cm gap as the maximum distance that allows the use of the mesh with a direct suture with additional release to advance the terminal edges of the *gluteus medius*.

The size of the displacement plays a very important role in the mechanics of the abductor apparatus. When the gap between muscles is more than 2.5 cm the surgeon must release the *gluteus medius* and reposition it in its anatomical position.

The number of surgical revisions did not statistically affect postoperative scores in our series.

In the postoperative follow-up, the Trendelenburg sign was absent in 75% of the patients. The Trendelenburg test with knee flexed and stair-climbing test were negative in 60% of the cases. Also the lateral position test and the lateral position with knee flexed test were negative in 50% of the population studied. In the lateral position, bending the knee and on climbing stairs, the role of tensor *fascia lata* was reduced. The decrease in these test values is through the mesh which was fixed in the muscle distally.

Although the abductor muscles functional test is demanding and both the injuries and the reconstruction that was performed were complex, the result was successful. Group A (<2.5 cm) and group B (>2.5 cm) had statically significant differences in their results for the Trendelenburg test with knee flexed and the stair-climbing test (*p* < 0.001). This is because when the knee is bent the *fascia lata* is relaxed, thus disabling its compensatory mechanism. Even after repair with Prolene^®^ mesh there may not be enough tension for the abductor mechanism to work properly with the knee bent.

The Trendelenburg test and its variations were negative in type V patients after their abductor mechanisms had been put back into place. This is because the abductor apparatus in these patients was continuous between the *gluteus medius* and *vastus lateralis*, always with a bone fragment of the greater trochanter. On repositioning the abductor mechanism in its anatomical location, Trendelenburg sign disappears.

According to our experience, whether the tear of the abductor mechanism is total or partial does not significantly affect repair results. Patients can be best classified in three groups: group A < 2.5 cm separation, group B > 2.5 cm separation, and group C continuous but located in the anterior position. According to Lübbeke et al. [4], after opening the deep fascia, we noted in all cases a “bald” trochanter with complete detachment of the *gluteus medius* muscle, after hip replacement surgery. Lübbeke et al. placed four or five nonabsorbable Bunnell-type sutures in the tendon ends. A 2.5-mm drill bit was used to make four or five transosseous straight tunnels and Mayo needles were used to pass the previously placed sutures through the tunnels and tie them. Results showed 31.5% of patients with no limp and 61% with substantial pain improvement [4]. Weber and Berry [9] also used tunneling with drill holes, refreshing of the bed, passing the sutures through the holes and tying the tendon. The follow-up was five years, and only five out of nine patients improved their limps. Rao et al. [5] published a technique in which 2 mm drill holes were made in the anterior part of the greater trochanter as close as possible to the normal insertion site, depending on the displacement of the gluteus muscle. Then #5 transosseous and Krakow stitch were used to attach the greater trochanter to the gluteus via three to five tunnels, depending on muscle bulk and conjoint tendon augmentation with a cellular human dermal matrix allograft (Graft Jacket^®^ Wright, Memphis, USA) [12]. The Trendelenburg sign became negative in 11 of 12 patients [5].

Beck et al. [22] described in three patients an 8 cm proximal advancement of the *m. vastus lateralis* without injury to the neurovascular pedicle and showed that the proximal advancement of the *m. vastus lateralis* can successfully bridge defects of the *gluteus medius* and partially restore abductor function. Kohl et al. [23] described the *m. vastus lateralis* shift technique to repair hip abductor defects without damaging the neurovascular pedicle in 11 patients with two-year follow-up and 27% of complications.

Miozarri et al. [24] published the late repair of abductor avulsion after the transgluteal approach and showed that aggressive repairs of dehiscence in abductor aponeurosis did not restore normal abductor muscle anatomy. MRI or direct examination demonstrated failed repairs in a third of the patients due to fatty degeneration in the anterior segments of the *gluteus medius* muscle, not reversible in 25% of the patients.

Whiteside [6] described the technique of transfer of the anterior portion of the gluteus maximus muscle to the greater trochanter and lateral femur, which compromises the major gluteus functioning as hip extensor, in 11 patients, with 16-month follow-up, and 90% success rate.

A different technique proposed is the use of Achilles tendon with a calcaneus bone block bank allograft [3] used in seven patients with a two-year follow-up. The Achilles tendon allograft attached to the greater trochanter produced relief of pain, increased abductor muscle strength, decreased limp, and brought about improvements in the Trendelenburg sign postoperatively. Domb et al. [25] proposed the endoscopic treatment and published series of 15 patients six with a partial tear, with a minimum two-year follow-up and reported good and excellent scores in 14 of 15 patients. Also McCormick et al. [26] report good results after endoscopic treatment in nine out of ten patients at one-year follow-up. Endoscopic treatment has its limitations when the gap in the gluteus medius muscle is large, or when it is displaced specially after several hip revision surgeries.

Amstutz and Maki stated that 5% of the hip showed separation and migration of the trochanteric fragments after total hip replacement using the trochanteric approach and a cruciate two wire technique of treatment. The abductor weakness was correlated with the amount of separation, especially if it exceeded 2 cm, the distance that the fragment migrated proximally would have mechanical effects that influence the weakness [27]. They used a wire mesh to help bind the fragmented or osteoporotic trochanter to the femoral shaft, but with activity, fatigue, and fragmentation of the mesh, this lead to an increased incidence of bursitis [27].

Repair of the abductor mechanism with Prolene^®^ mesh (©Ethicom, Johnson & Johnson, NJ, USA) has proven effective for both partial and total lesions. It does not affect the insertion of the external vastus muscle or the insertion of the gluteus medius muscle when the lesion has a gap of less than 2.5 cm. However, when the gap is wider than 2.5 cm, the gluteus medius muscle must be released to move it closer to the external vastus and thus reduce the existing gap. This surgical maneuver to rotate the muscle does not produce a vascular tissue damage, because it does not affect the blood supply of the rotated muscle. The treatment is simple, maintaining continuity between the *gluteus medius* and *vastus lateralis* with the *tensor fascia lata* by the mesh, minimizing the possible gap between them, and without setting this reconstruction to the greater trochanter. It is really a new concept, to maintain the continuity between the gluteus medius and the *vastus lateralis* tightening the *fascia lata* by the mesh without fixing it to the femur.

The weakness of this study is that we only evaluated the functional results of the abductor mechanism repair, and it was a small population series.

The strength is that we demonstrated that the mechanism can be repaired by a simple procedure.

## Conflict of interest

The authors declare no conflict of interest in relation with this paper.
